# Does the Use of Different Types of Probiotics Possess Detoxification Properties Against Aflatoxins Contamination in Rabbit Diets?

**DOI:** 10.1007/s12602-022-09990-w

**Published:** 2022-09-26

**Authors:** Said I. A. Mohamed, Sabry A. M. Shehata, Sabry M. Bassiony, Samir A. M. Mahgoub, Mohamed E. Abd El-Hack

**Affiliations:** 1 Animal Production Department, Faculty of Agriculture, Zagazig University, Zagazig, 44511 Egypt; 2 Agricultural Microbiology Department, Faculty of Agriculture, Zagazig University, Zagazig, 44511 Egypt; 3 Department of Poultry, Faculty of Agriculture, Zagazig University, Zagazig, 44511 Egypt

**Keywords:** Aflatoxins, Probiotics, Degradation, Performance, Blood, Rabbits

## Abstract

The present work was carried out to study the ability of five probiotics on the in vitro degradation of Aflatoxins B_1_ (AFB_1)_. The best results of in vitro were tested on the detoxification of AFB_1_ in rabbits. A total of 40 growing New Zealand White (NZW) male rabbits were assigned to five experimental groups. Animals were fed the following diets: basal diet (control), basal diet contaminated with 300 ppb AFB_1_, basal diet contaminated with 300 ppb AFB_1_. + probiotic 3 (0.5 g/kg diet), basal diet contaminated with 300 ppb AFB_1_ + ajowan (0.5 g/kg diet), and basal diet contaminated with 300 ppb AFB_1_ + probiotic 3 (0.5 g/kg diet) + ajowan (0.5 g/kg diet). Live body weight significantly (*P* < 0.05) decreased in rabbits fed AFB_1_ contaminated diet compared to the control rabbits. All additives improved (*P* < 0.05) the live body weight. The best improvement occurred with probiotics + ajowan. The addition of probiotics increased (*P* < 0.05) daily body weight gain in all weeks except the first week. Adding ajowan or ajowan + probiotic led to a significant (*P* < 0.05) increase in live body weight gain and feed intake compared to rabbits fed AFB_1_ alone. The digestion coefficients of dry matter (DM), organic matter (OM), crude fiber (CF), ether extract (EE), nitrogen-free extract (NFE), and digestible crude protein (DCP) significantly (*P* < 0.05) decreased in rabbits fed AFB_1_ contaminated diet. All additives improved (*P* < 0.05) the digestibility coefficients of DM, OM, EE, CF, NFE, and total digested nutrients (TDN)%. The best improvement occurred with probiotics + ajowan. Concentrations of serum total protein, albumin and globulin significantly (*P* < 0.05) decreased in rabbits fed AFB_1_ contaminated diet compared with the control rabbits. In conclusion, the addition of probiotic 3 (AVI-5-BAC) + ajowan could be recommended to eliminate the toxicity of AFB1 and improve growth performance criteria in rabbits.

## Introduction

The Food and Agricultural Organization (FAO) indicated that mycotoxins contaminate 25% of global feedstuffs following the current mycotoxin limits set by the European Union (EU) and the Codex Alimentations. However, 60 to 80% of foods have measurable mycotoxin levels [1]. The contamination of complete feeds in Europe with aflatoxins (AFs) varied greatly throughout the previous 10 years. The percentage of AFB1-positive feed samples between 2006 and 2007 was 8%, with the mean contamination being 47 mg/kg and the highest contamination being 311 mg/kg [2]. Aflatoxin, zearalenone (ZEA), deoxynivalenol (DON), ochratoxin A (OTA), patulin, fumonisins, T-2, and ergot alkaloids are among the mycotoxins that are regularly regulated by law in the European Union. But AFs were more frequent. The fungus *Aspergillus flavus* and *Aspergillus parasiticus* create AFs, secondary toxic compounds that contaminate numerous feedstuffs and cause major health issues in both people and animals [3]. Although they were naturally present in milk, AFs species are named based on their Green and Blue fluorescence characteristics in thin layer chromatography (TLC) (B1, B2, G1, G2, M1, M2) Meulenaer [63].

During growth, on feeds, and in foods, several strains of *Aspergillus flavus* and *Aspergillus parasiticus* produce a category of poisonous and cancer-causing secondary metabolites known as AFs. Infesting both living and dead plants and animals, the fungus spores can be found in the air and soil all over the planet. The content, total excretion, and carry-over of aflatoxin B1 (AFB1) into milk as aflatoxin M1 (AFM1) were the subjects of an experiment [4]. A total of 550,000–600,000 new cases are reported annually [5]. The food and feed industries suffer considerable financial losses due to mycotoxin contamination, posing a serious public health hazard. AFs have hazardous (carcinogenic, teratogenic, and mutagenic) properties that can harm human and animal health [6]. Strong mycotoxin AFB1 has mutagenic, carcinogenic, teratogenic, hepatotoxic, and immunosuppressive traits [7]. Because eating foods contaminated with mycotoxins can have various negative health impacts on humans and animals, mycotoxin contamination of agricultural goods is a big issue worldwide [8].

The rabbit is one of the species most vulnerable to the harmful effects of AF. Consuming diets contaminated with AFs caused anorexia, decreased feed intake, altered feed utilization, reduced weight gain, inhibited growth, immune suppression, increased susceptibility to various stressors and diseases, increased mortality rate, altered reproductive performance, and ultimately led to financial issues for the livestock and poultry industries [9].

Ajowan (*Trachyspermum ammi* L.), an annual herb with roots in the Middle East, presumably in Egypt and the Indian subcontinent but also in Iran and Afghanistan, is a member of the Apiaceae (Umbelliferae) family of plants [10–13]. Ajowan contains a substance with known antibacterial, antifungal, antihelminthic, and antiseptic properties [14]. Phenols, particularly thymol and carvacrol, which are significant pharmacologically active chemicals, are among the principal active components of ajowan [15–18]. The present study hypothesized that probiotics and/or ajowan may eliminate the toxicity of AFB1 and improve growth performance of rabbits. The current research aimed to determine how well five different probiotics might break down AFB_1_ (in vitro). The best results were tested on the detoxification of AFsB_1_ in rabbits.

## Materials and Methods

The current research aimed to examine the potential of five probiotics to degrade AFB_1_ at the Rabbit Farm and Laboratories of the Animal Production Department, Faculty of Agriculture, Zagazig University, Zagazig, Egypt (in vitro). Additionally, the finest probiotic and ajowan were used to detoxify AFB_1_ in growing rabbits.

### In Vitro Study

#### Probiotics

1. First probiotic (Biogen S), each 1 kg contained: *Bacillus subtilis* natto not less than 1 × 10^11^ CFU. SAMU MEDIAN CO. LTD, China, manufactured this product.
2. Second probiotic (Promax), each kg contained: *Lactobacillus acidophilus* 150 g (5 × 10^9^ CFU), *Lactobacillus plantarum* 500 g (5 × 10^8^ CFU), vitamin A (8,000,000 IU), vitamin B_1_ (600 mg), vitamin B_2_ (1500 mg), vitamin C (38,000 mg), vitamin D_3_ (1,500,000 IU), vitamin E (4000 mg), vitamin K_3_ (2000 mg), pantothenic acid (12,000 mg), nicotinic acid (12,000 mg), potassium citrate (40 g), sodium chloride (33 g), magnesium sulfate (60 g), dextrose up to (1000 g). Egyptian European Co. produced this product for Vet. Industries (EMIC VET).
3. Third probiotic (AVI-5-BAC), each g contained: *Lactobacillus acidophilus* 10 g (1 × 10^8^ CFU), *Lactobacillus plantarum* 5 g (9.8 × 10^7^ CFU), *Bifidobacterium bifidum* 5 g (2 × 10^6^ CFU) and maltodextrin add to 1 kg. SURE PHARMACEUTICA, USA, produced this product.
4. Fourth probiotic (YEAST PLUS), each 1 kg contained: *Saccharomyces cerevisiae* (250,000 mg), vitamin D3 (2,000,000 IU), Dl methionine (10,000 mg), selenium (200 mg), calcium carbonate up to 1000 g. This product was produced by United Brothers for Feed Supplements, Egypt.
5. Fifth probiotic (GUARDIZEN-M), each 1 kg contained: *Lactobacillus plantarum* (1.2 × 10^6^ CFU/g), *Lactobacillus bulgaricus* (1.2 × 10^6^ CFU/g), *Lactobacillus rhamnosus* (1.2 × 10^6^ CFU/g), *Lactobacillus acidophilus* (1.2 × 10^6^ CFU/g), *Bifidobacterium bifidum* (1.2 × 10^6^ CFU/g), *Streptococcus faecium* (1.2 × 10^6^ CFU/g), *Enterococcus faecium* (1.2 × 10^6^ CFU/g), *Aspergillus oryzae* (1.2 × 10^6^ CFU/g), *Candida pintolopesii* (1.2 × 10^6^ CFU/g), carrier dextrose (994.4 g). This product was produced by DONC BNC CO. LTD, South Korea.

#### Screening the Ability of Probiotics on the Degradation of AFB1 by Thin Layer Chromatography (TLC) Analysis

The standard of AFs from the Regional Centre for Food and Feed, Ministry of Agriculture, Giza, Egypt, was graciously contributed by Dr. Khaled El-Meligy; 200 ppb of standard AFB1 were obtained by dissolving it in a solution of methanol and water (2:8). The Market of Veterinary Medicine was used to obtain the probiotics, which were then grown in nutritional broth with or without AFB1. The treatments were:

1. Culture (20 ml) + AFB_1_ (1 ml)
2. Culture (20 ml) + AFB_1_ (1 ml) + probiotic 1
3. Culture (20 ml) + AFB_1_ (1 ml) + probiotic 2
4. Culture (20 ml) + AFB_1_ (1 ml) + probiotic 3
5. Culture (20 ml) + AFB_1_ (1 ml) + probiotic 4
6. Culture (20 ml) + AFB_1_ (1 ml) + probiotic 5

The treatments were incubated at 37 °C for 72 h, after which 20 ml of each treatment was extracted using 20 ml chloroform. This mixture was then thoroughly agitated for 5 min, transferred to a separatory funnel, allowed to stand, and drained the bottom layer into a clean flask. The chloroform extracts were dried by evaporation, and AFs were found by TLC.

#### Preparing the Thin Layer Chromatography (TLC) Plates

TLC Plates Were Prepared [19] as Follows:

To prevent air bubbles, 10 g of silica gel (GF 254) was aggressively mixed with 30 ml of warm distilled water. Chromatographic glass plates (20 × 20 cm) were air dried after being promptly covered with 0.05 mg of silica gel dispersion. The plates were heated in an electric oven for 1–2 h at 110–120 °C to activate them. Plates were taken out of the oven immediately to cool in a desiccator.

Spotting on TLC:

A predetermined volume of chloroform was used to dissolve the residual from purified extracts (0.5 ml). On TLC plates against standard AFB1, micropipettes spotted the concentrated crude extract’s known volume (100 μl). Spots were kept uniformly small and small in size. Plates were spotted and then left to air dry.

Development of Solvents:

Following AOAC (1980), an appropriate solvent system (chloroform:acetone (90:10v/v)) was placed in a jar. Plates were taken out of the jar and set vertically in the air until dry after the solvent system had migrated about 16 cm. The plates were carefully inserted into the jar.

Detection of Aflatoxins by TLC [19]:

After development, the plates were allowed to air dry before being examined under long-wavelength (366 nm) UV light to compare the color intensity of the spots to the standard. The distinctive fluorescent dots are present at the same Rf levels as the common toxin. Each poison was presumed to exist based on UV excitation. The TLC technique and UV spectrophotometer were used to calibrate the toxin concentration in accordance with the AOAC [19] method for qualitative toxin emission.

### In Vivo Study

The best probiotic of in vitro (probiotic 3) and ajowan was tested on detoxification of AFB_1_ in rabbits.

#### Preparation of Aflatoxin B_1_

To create AFB1, *Aspergillus flavus* MD 341 was obtained from the Dokki, Egypt-based Central Laboratory of Residues of Analysis of Pesticides Heavy Metals in Foods. On liquid media containing 2% yeast extract and 20% sucrose, the fungus was cultured for 8 days. The media was sprayed into a pelleted diet (300 ppb of AFB1). A reversed-phase column was used in the extraction, filtration, and quantitative HPLC analysis of aflatoxins [19]. The mobile phase contained 45% methanol and was injected into the apparatus at a flow rate of 1 ml per minute. A fluorescence detector was used to find analyses, and the column temperature was set to 40 °C. Aflatoxin was bought from Sigma-Aldrich (ASA). The media was discovered just to contain AFB1.

#### Treatments

In this experiment, 40 growing New Zealand White (NZW) male rabbits with an average body weight of 800 ± 120 g were assigned to 5 experimental groups (8 animals/each). The animals in experimental groups were fed the following diets:

1. Basal diet (Control).
2. Basal diet contaminated with 300 ppb AFB_1_.
3. Basal diet contaminated with 300 ppb AFB_1_. + probiotic 3 (0.5 g/kg diet)
4. Basal diet contaminated with 300 ppb AFB_1_. + ajowan (0.5 g/kg diet)
5. Basal diet contaminated with 300 ppb AFB_1_. + probiotic 3 (0.5 g/kg diet) + ajowan (0.5 g/kg diet).

The ajowan was purchased from the local market, fine ground, and added to ingredients before pelleting. The ingredient (%) and chemical composition of the basal diet are shown in Table 1.

**Table 1:** Ingredients and chemical composition of diets fed to rabbits

Items	%
*Ingredients*	
Yellow corn	17.00
Clover hay	35.00
Wheat bran	20.00
Barley	10.00
Soybean meal	13.00
Molasses	3.00
Sodium chloride	0.10
Methionine	0.30
Vitamins and minerals premix	0.30
Bone meal	1.00
Limestone	0.30
Total	100
*Chemical composition (DM) basis*	
Dry matter	100
Organic matter	87.53
Crude protein	19.80
Crude fiber	12.39
Ether extract	2.58
Nitrogen free extract	52.76
Ash	12.47

#### Rabbits Rearing

Each animal was kept in its stainless steel cage. For the trial, all rabbits were kept in the same management, sanitary, and environmental circumstances with constant access to fresh water (8 weeks). Rabbits were fed ad libitum during the whole experiment. At the start of the trial and weekly intervals throughout the experiment, each rabbit was weighed separately. Before the animals had access to food and water, the weight was taken. The gain in body weight was calculated. Additionally, feed intake was daily calculated after being determined weekly. It was determined what the feed conversion ratio was (feed intake/weight gain).

#### Digestibility Trials

Digestibility trials were conducted over 5 days. Digestibility tests were conducted to assess the impact of treatments on nutritional digestibility and feeding values such as TDN percent and DCP percent after the study period (8 weeks). Throughout the collection period, samples from each animal’s dried feces and provided meals were collected daily for chemical analysis in accordance with AOAC [20].

#### Blood Analysis

In a private medical lab, blood hematological parameters were conducted. Blood samples from four groups of rabbits were taken at the time of slaughter at the end of the experimental feeding period to evaluate various blood parameters. Using commercial kits acquired from Diamond Diagnostics Company, Egypt, it was possible to assess the levels of total protein, albumin, aspartate and alanine aminotransferases (AST and ALT), alkaline phosphatase (ALP), total protein, and albumin in rabbit blood serum.

#### Statistical Analysis

SAS’s (1996) general linear model program was used to examine the experiment’s data statistically. Duncan’s Multiple Range Test evaluated if there were significant changes between treatment means [21]. The statistical model used was:

$${Y}_{ij}=\mu +{T}_{i}+{e}_{ij}$$

where *Y*_*ij*_ = observed value; *µ* = overall mean; *T*_*i*_ = treatment effect (control, and 1–6); and *e*_*ij*_ = random error. Differences among recorded means were estimated by the test of Student–Newman–Keuls. The SEM and mean values were reported. The differences between groups are considered significant at *P* < 0.05.

## Results and Discussion

### In Vitro Study

#### Degradation of Aflatoxin B_1_ by Probiotics Using Thin Layer Chromatography (TLC)

**Fig. 1:**
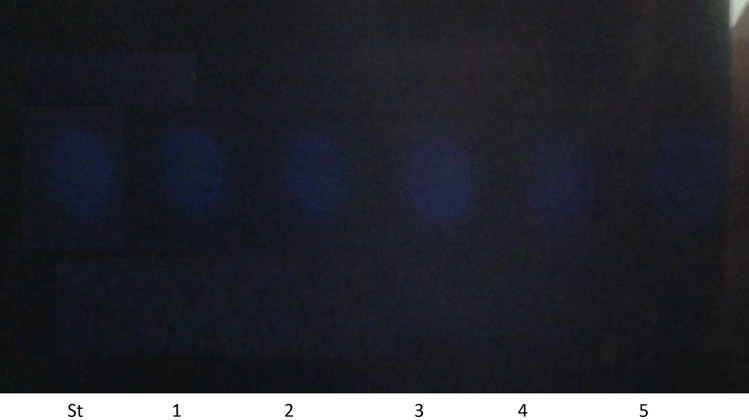
TLC analysis of aflatoxin B_1_ degradation by probiotics

All types of probiotics could degrade AFB1 (Table 2 and Fig. 1). Probiotic 3 performed the AFB1 degradation process the best. These findings concur with Atya [22], who used TLC to examine the impact of 43 bacterial and 10 fungal species on the degradation of AFs. There were two fungi and nine bacterial isolates that could degrade down AFs. These isolates were collected for further High-Performance Liquid Chromatography examination (HPLC). According to HPLC data, three bacterial and one fungal isolate destroyed AFs by more than 90%.

**Table 2:** Degradation of aflatoxin B_1_ by probiotics

Probiotic no	TLC result
1	50%
2	50%
3	70%
4	65%
5	40%

Since many mycotoxins are chemically stable but do not seem to accumulate in natural surroundings, biological degradation of mycotoxins happens in nature. Therefore, environmental samples rich in microorganisms were chosen as sources for choosing microorganisms that degrade AFB1, such as animal feces, degraded barks, soils, and cereal grains [23].

It has been noted that a number of bacteria bind or degrade AFs in foods and feeds. This study tested the ability of 20 lactic acid bacteria (LAB) strains and bifidobacterial to bind AFB1 from contaminated solution. Twelve Lactobacillus, five Bifidobacterium, and three Lactococcus strains were chosen for usage in the food sector. According to the findings, these strains bind between 5.6 and 79.7% of AFB1 from the solution. Two strains of *Lactobacillus amylovorus* and one strain of *Lactobacillus rhamnosus* eliminated more than 50% of AFB1 Peltonen [66].

AFB1, a chemical with a modified furan and lactone ring, was bio-transformed by *Pseudomonas putida* into three new compounds with distinct structural properties (AFD1, AFD2, and AFD3). The percentage of AFs that were bound by LAB ranged from 19.41 to 75.06%. The AF-binding activity displayed a time-dependent pattern when different incubation times were considered. During the investigated course of incubation durations, *Lactobacillus rhamnosus* TMU094 bound 25.64 to 75.06%, *Lactobacillus fermentum* bound 38.63 to 72.15%, *Pediococcus pentosaceus* bound 24.86 to 63.21%, and *L. rhamnosus* PTCC1637 bound 19.41 to 35% of AFB1. These findings demonstrated the capability of native LAB strains to bind AFB1 [24] efficiently. According to toxicity research conducted on HeLa cells, the newly synthesized chemicals are less hazardous than AFB1 [25].

AFB1 was degraded by the chicken cecum-derived AFB1-degrading bacteria CG1061 at a rate of 93.7% by HPLC, which was isolated and characterized. A multiplex PCR assay and examination of the 16S RNA gene sequence revealed that CG1061 was a non-pathogenic strain of *Escherichia coli*. The *E. coli* CG1061’s culture supernatant demonstrated a 61.8% disintegration rate. The active component was constitutively released into the extracellular area, as evidenced by the intracellular extracts’ low degradation rate of only 17.6% [26].

### In Vivo Study

#### Effect of Aflatoxin B_1_ on Growth Performance of Rabbits

Tables 3, 4, 5, and 6 indicate how ajowan and the probiotic aflatoxin B1 (AFB1) affect body weight, daily body gain, feed intake, and conversion. When compared to control rabbits, the live body weight of rabbits fed an AFB1-contaminated food declined significantly (*P*<0.05) from the third week through the end of the experiment (8 weeks). The live body weight was improved by all supplements (probiotic, ajowan, and probiotic + ajowan) (*P*<0.05). The combination of probiotics and ajowan produced the best results. At all weeks of the trial period, the AFB1 diet decreased feed consumption and daily body weight gain (*P*<0.05). In all weeks but the first, adding probiotics increased (*P*<0.05) the daily increase in body weight.

**Table 3:** Effect of treatments on body weight of rabbits

Items	Treatments				
	Control	Aflatoxin	Aflatoxin + probiotic	Aflatoxin + ajowan	Aflatoxin + probiotic + ajowan
Initial body weight	808.5 ± 139.94	800 ± 110.23	800.62 ± 139.45	815.25 ± 130.14	773.13 ± 138.69
1 week	1052.87 ± 118.79	982.5 ± 136.04	1000.61 ± 135.18	1043.38 ± 143.14	993.75 ± 134.82
2 weeks	1275.15 ± 123.54	1135.66 ± 134.27	1196.26 ± 129.28	1245.89 ± 157.09	1208.09 ± 117.23
3 weeks	1503.94 ± 129.73a	1293.51 ± 127.04b	1419.42 ± 128.34a	1442.17 ± 164.13a	1450.01 ± 126.22a
4 weeks	1730.67 ± 142.58a	1427.14 ± 133.61b	1628.51 ± 137.71a	1645.94 ± 180.13a	1682.06 ± 146.13a
5 weeks	1923.31 ± 143.1a	1557.48 ± 125.86b	1798.54 ± 151.23a	1831.58 ± 190.48a	1915.79 ± 141.8a
6 weeks	2109.58 ± 158.28a	1695.67 ± 125.98b	1969.78 ± 147.16a	2045.29 ± 182.37a	2120.12 ± 140.89a
7 weeks	2321.99 ± 173.04a	1842.55 ± 133.23b	2167.27 ± 177.4a	2258.44 ± 191.1a	2329.49 ± 123.19a
8 weeks	2556.95 ± 166.88a	1987.51 ± 127.47c	2358.25 ± 178.3b	2459.06 ± 183.16ab	2533.89 ± 125.71a

a, b, c means in the same row with different superscripts are significantly (*P* < 0.05) different

**Table 4:** Effect of treatments on body weight gain of rabbits

Items	Treatments				
	Control	Aflatoxin	Aflatoxin + probiotic	Aflatoxin + ajowan	Aflatoxin + probiotic + ajowan
1 week	34.91 ± 5.06a	26.07 ± 3.23c	28.57 ± 4.08bc	32.59 ± 2.71ab	31.52 ± 6.6ab
2 weeks	31.75 ± 2.13a	21.88 ± 2.16b	27.95 ± 4.03a	28.93 ± 4.18a	30.62 ± 5.63a
3 weeks	32.68 ± 4.43a	22.55 ± 1.98c	31.88 ± 4.27a	28.04 ± 4.4b	34.56 ± 2.35a
4 weeks	32.39 ± 7.11a	19.09 ± 2.41b	29.87 ± 3.72a	29.11 ± 3.3a	33.15 ± 3.31a
5 weeks	27.52 ± 4.4 b	18.62 ± 4.15c	24.29 ± 0.37b	26.52 ± 3.06b	33.39 ± 4.45a
6 weeks	26.61 ± 3.91ab	19.74 ± 4.2c	24.46 ± 1.86b	30.53 ± 3.39a	29.19 ± 4.6a
7 weeks	30.34 ± 2.37a	20.98 ± 3.33b	28.21 ± 5.9a	30.45 ± 4.43a	29.91 ± 4.81a
8 weeks	33.57 ± 3.54a	20.71 ± 2.13c	27.28 ± 1.5b	28.66 ± 3.16b	29.2 ± 2.71b

a, b, c means in the same row with different superscripts are significantly (*P* < 0.05) different

**Table 5:** Effect of treatments on feed intake of rabbits

Items	Treatments				
	Control	Aflatoxin	Aflatoxin + probiotic	Aflatoxin + ajowan	Aflatoxin + probiotic + ajowan
1 week	90.00 ± 5.55a	80.00 ± 9.53b	95.00 ± 9.15a	97.00 ± 3.78a	95.38 ± 10.23a
2 weeks	95.00 ± 1.85a	85.00 ± 2.93b	98.75 ± 9.82a	95.00 ± 4.5 a	94.00 ± 7.25a
3 weeks	100.00 ± 3.34a	90.00 ± 1.2b	103.00 ± 8.45a	105.00 ± 7.35a	105.00 ± 7.19a
4 weeks	125.0 ± 13.98b	93.00 ± 4.24c	135.00 ± 10.69ab	140.00 ± 9.87a	135.00 ± 10.32ab
5 weeks	127.00 ± 4.54b	100.00 ± 5.29c	130.00 ± 5.37ab	135.00 ± 4.84a	130.00 ± 6.14ab
6 weeks	130.00 ± 8.73a	107.00 ± 13.89b	127.00 ± 4.31a	130.00 ± 6.05a	135.00 ± 9.07a
7 weeks	133.00 ± 8.4a	121.25 ± 5.37b	135.00 ± 6.2a	131.75 ± 8.08a	137.0 ± 8.45a
8 weeks	140.00 ± 5.58a	110.00 ± 5.15b	140.00 ± 5.29a	143.00 ± 3.78a	140.00 ± 4.63a
Average	117.5 ± 19.34a	98.28 ± 13.89b	120.47 ± 18.37a	122.09 ± 19.77a	121.14 ± 19.75a

a, b, c means in the same row with different superscripts are significantly (*P* < 0.05) different

**Table 6:** Effect of treatments on feed conversion of rabbits

Items	Treatments				
	Control	Aflatoxin	Aflatoxin + probiotic	Aflatoxin + ajowan	Aflatoxin + probiotic + ajowan
1 week	2.57 ± 0.25 c	3.07 ± 0.24 ab	3.33 ± 0.35 a	2.97 ± 0.32 b	3.03 ± 0.37 ab
2 weeks	2.99 ± 0.24 c	3.88 ± 0.34 a	3.53 ± 0.32 b	3.28 ± 0.46 bc	3.07 ± 0.44 bc
3 weeks	3.06 ± 0.54 b	3.99 ± 0.36 a	3.23 ± 0.22 b	3.74 ± 0.39 a	3.04 ± 0.31 b
4 weeks	3.85 ± 0.76 b	4.87 ± 0.53 a	4.51 ± 0.28 ab	4.80 ± 0.43 a	4.07 ± 0.53 b
5 weeks	4.61 ± 0.72 b	5.37 ± 0.77 ab	5.35 ± 0.5a	5.09 ± 0.65ab	3.89 ± 0.37c
6 weeks	4.88 ± 0.64ab	5.42 ± 0.64a	5.19 ± 0.47ab	4.25 ± 0.38c	4.62 ± 0.52bc
7 weeks	4.38 ± 0.26b	5.76 ± 0.92a	4.79 ± 0.92b	4.32 ± 0.45b	4.58 ± 0.57b
8 weeks	4.17 ± 0.51b	5.31 ± 0.67a	5.13 ± 0.3a	4.98 ± 0.47a	4.79 ± 0.34a
Average	3.81 ± 0.8a	4.71 ± 0.97c	4.38 ± 0.91b	4.16 ± 0.8b	3.88 ± 0.76a

a, b, c means in the same row with different superscripts are significantly (*P* < 0.05) different

In addition, as compared to rabbits fed AFB1 alone, adding ajowan or ajowan + probiotic increased live body weight gain and feed consumption significantly (*P*<0.05). The daily feed intake increased (*P* 0.05) over all weeks with the addition of probiotics. Rabbits fed a diet contaminated with AFB1 had the worst feed conversion. Probiotic plus ajowan or ajowan + probiotics were added, and this resulted in a substantial (*P*<0.05) increase in feed conversion (Table 6).

According to Shehata [27, 44], Somorin et al. [9], Helal [28], Sorour [29], and Yang et al. [30], the performance of the rabbits is consistent with their findings. The depression in feed intake, reduction in protein, lipid, and carbohydrate metabolism, and decreased absorption of dissolved vitamins in lipid may all contribute to AFB1’s decreased growth performance [31, 9]. Through the stimulation of the generation of reactive oxygen species (ROS) or the enhancement of tissue sensitivity to peroxidation, the mycotoxins directly cause lipid peroxidation. Because AFs encourage the enzymatic production of intracellular ROS like the superoxide anion, which in turn causes the AF metabolite to bind to DNA, RNA, and proteins, AFs are hazardous. The expression of inflammatory response-related proteins in the liver, such as NFKB1 and GPX1, can also rise as a result of AF consumption, according to Pate et al. [65].

Probiotics’ findings concur with those of Wang et al. [26, 32], Samuel et al. [25], and Fashandi et al. [62]. Probiotics may operate as biodegradable toxins that can act as an antioxidant by triggering the creation of enzymes, which in turn causes weight gain by enhancing protein metabolization and vitamin and mineral absorption [33], Abd El-Aziz et al. [34, 55], Nasr [64]. Supplementing with biodegrading agents affects the digestive tract, promoting the production of digestive enzymes that are essential for improved digestion and, consequently, weight gain Dersjant et al. [60]. By lengthening the villi, decreasing intestinal pH, eliminating intestinal bacteria, increasing the secretion of auxiliary digestive enzymes, and improving nutrient absorption, biodegradation agent supplementation promotes growth [35]. Ajowan’s findings are consistent with those of Hajare et al. [36], who discovered that the aqueous extract of ajowan seeds contained an AF inactivation component. Over the controls, a roughly 80% decrease in the overall amount of AFs was seen.

Additionally, these results support Iram et al. [37]. They stated that in vitro and in vivo tests were performed to see whether an aqueous extract of ajowan seeds and leaves could detoxify AFB1 and AFB2. The AFB1 and AFB2 were shown to be significantly (*P*<0.05) degraded by ajowan seeds extract, by 92.8 and 91.9%, respectively. The extract from ajowan leaves, however, performed less well at destroying these AFs. Eight degradation products of AFB1 and AFB2 were generated according to the structural study of the toxin by LCMS/MS. By removing the double bond from the terminal furan ring and changing the lactone group, most of the products were created, suggesting they were less dangerous than the parent compounds. The minimal toxicity of degradation products was further supported by brine shrimp bioassay, demonstrating that ajowan seeds extract can be a powerful detoxification agent for AFs. Ajwoan’s therapeutic effects are also mentioned, including its 40% thymol content and properties as an antispasmodic, stimulant, tonic, and carminative. It is prescribed for cholera and given for diarrhea, atonic dyspepsia, and flatulence. Presence of various phytochemical components, including volatile oils, phenolic compounds, minerals, proteins, fats, carbohydrates, glycosides, and fiber [10–12]. Antioxidant, antibacterial, antifungal, hypolipidemic, antihypertensive, antispasmodic, bronchodilator, diuretic, antitussive, anthelmintic, and abortifacient are only a few examples of the many pharmacological qualities [38–41].

To efficiently produce high-quality meat without antibiotics, ajowan can be promoted as a non-antibiotic growth promoter (NAGP) in the broiler sector [42]. Latter authors examined how ajowan affected the performance of broiler chicks. The basal diet (control group), the basal diet plus 0.02% ajowan powder, and the basal diet plus 0.02% virginiamycin powder were given to the chicks. Data indicated that feed consumption significantly increased in treated groups compared to controls. Additionally, there was a substantial (*P*<0.05) increase in total body weight and body weight gain in the treated groups Dinodiya [12, 61].

#### Digestion Coefficients and Nutritive Values of the Experimental Diets

Effect of Aflatoxin B_1_ (AFB_1_):

Comparing rabbits fed an AFB1-contaminated diet to control rabbits, the digestion coefficients of DM, OM, CF, EE and NFE and nutritional values as TDN and DCP were considerably (*P*<0.05) decreased (Table 7). The detrimental effects of AFB1 on nutritive values and digestibility are consistent with those described by Salem et al. [43], Shehata (27, 44, and Helal [28]. AFB1 may interfere with the utilization of dietary nutrients, which would explain its negative impact on nutrient digestibility [45]. The digestibility coefficients of DM, OM, EE, CF, and NFE and TDN percent were improved (*P*<0.05) by all additions (probiotic, ajowan, and probiotic + ajowan). Probiotics plus ajowan caused the greatest improvement (Table 7).

**Table 7:** Effect of treatments on digestion coefficient and nutritive values

Items	Treatments				
	Control	Aflatoxin	Aflatoxin + probiotic	Aflatoxin + ajowan	Aflatoxin + probiotic + ajowan
**Digestion coefficient (%)**					
DM	75.57 ± 1.04a	64.27 ± 0.53c	71.29 ± 1.06b	72.11 ± 1.54b	72.59 ± 0.47b
OM	76.89 ± 1.35a	68.95 ± 5.27b	73.78 ± 1.15a	74.44 ± 1.71a	74.87 ± 0.72a
CP	81.10 ± 1.63a	78.11 ± 0.99b	78.21 ± 1.25b	78.74 ± 1.31b	80.00 ± 1.88ab
EE	85.89 ± 0.14a	74.15 ± 4.44c	80.39 ± 3.66b	79.84 ± 1.67b	82.10 ± 2.85ab
CF	41.99 ± 3.05a	29.19 ± 2.55b	38.82 ± 2.47a	38.91 ± 1.22a	40.46 ± 2a
NFE	83.06 ± 1.2a	70.97 ± 0.74c	80.01 ± 1.41b	81.04 ± 2.3ab	81.00 ± 0.72ab
**Nutritive values (%)**					
TDN	69.95 ± 1.35a	60.83 ± 0.95c	67.17 ± 1.11b	67.68 ± 1.53b	68.33 ± 0.73ab
DCP	16.06 ± 0.33a	15.47 ± 0.2b	15.50 ± 0.23b	15.59 ± 0.26b	15.82 ± 0.35ab

a, b, c means in the same row with different superscripts are significantly (*P* < 0.05) different

The results of the probiotic studies support those of Kasmani et al. [24]. They stated that *Lactobacillus plantarum* and *Lactobacillus paracasei* could successfully reduce the immunological toxicities of AFs in mice and that *Lactobacillus rhamnosus* could reduce the concentration of AFB1 by 44–54% utilizing a chicken intestinal loop approach. TLC and HPLC analysis revealed that nine bacterial isolates might degrade down AFs. According to HPLC detection, three bacterial cultures had AF degradation ratio greater than 90% [46].

Several bacteria identified from soil, dung, nuts, and other habitats, including *Rhodococcus erythropolis*, *Mycobacterium fluoranthenivorans*, *Stenotrophomonas maltophilia*, *Enterobacteriaceae* sp., *Myxococcusfulvus*, *Bacillus subtilis*, and *Pseudomonas putida*, efficiently degrade AFB1 [47, 48, 25].

AFB1 is eliminated from contaminated water by lactic acid bacteria (*Bifidobacterium angulatum*, *Lactobacillus acidophilus*, *L. rhamnosus*, *L. plantarum*, and *Streptococcus thermophiles*) [49]. As starter cultures, LABs from various origins, including Egypt, Thailand, and German culture collecting facilities, can be employed to lower AFM1. Eleven probiotic Lactobacillus strains were added, and the resulting AFM1 binding ability ranged from 4.13 to 64.16%. Additionally, the analysis of the stability of the bacterial-AFM1 complex revealed a minor release of AFM1 in the first and second washes but total stability in the third wash [50]. Ajowan’s increased digestibility and nutritional value may be a result of its advantageous effects on biological functions [41, 42]

#### Blood Biochemistry 

Table 8 illustrates how eating an AFB1-contaminated diet affected the blood biochemistry of rabbits. When compared to control rabbits, concentrations of total protein, serum albumin, and globulin significantly (*P*<0.05) decreased in rabbits fed an AFB1-contaminated diet (Table 8). On the other hand, the blood of rabbits fed an AFB1-contaminated diet had significantly (*P*<0.05) higher levels of the liver enzymes like AST, ALT, and ALP activities. The negative impact of AFB1 on blood components is consistent with the findings of Helal [28] and Sorour [29]. They found that NZW rabbits fed a diet containing AFB1 decreased serum total protein and albumin (*P*<0.05). Our findings on AST, ALT, and ALP enzyme activity are consistent with those of Yousef et al. [51] and Sorour [29]. Increased cell membrane permeability or hepatocellular necrosis may cause elevated ALT activity. Aflatoxin’s effects on protein synthesis and cellular integrity in the liver may be responsible for the decline in total protein and albumin levels [52]. The dangerous impact of aflatoxin on immunity may be to blame for the drop in globulin content [31].

**Table 8:** Effect of treatments on blood biochemistry of rabbits

Items	Treatments				
	Control	Aflatoxin	Aflatoxin + probiotic	Aflatoxin + ajowan	Aflatoxin + probiotic + ajowan
AST (u/l)	34.73 ± 1.62 b	56.26 ± 10.83 a	37.24 ± 1.91 b	40.71 ± 6.49 b	35.89 ± 2.54 b
ALT (u/l)	41.68 ± 1.46 b	68.55 ± 2.88 a	43.89 ± 2.45 b	41.89 ± 7.77 b	42.46 ± 2.32 b
ALP (u/l)	180.00 ± 3 c	264.50 ± 11.5 a	205.00 ± 6 b	191.00 ± 6 bc	180.00 ± 10.5 c
Total protein (g/dl)	6.83 ± 0.9 a	4.19 ± 0.6c	5.32 ± 0.21 b	5.36 ± 0.6 b	5.47 ± 0.24 b
Albumin (g/dl)	4.46 ± 0.9 a	3.32 ± 0.14 c	4.04 ± 0.9 b	4.04 ± 0.5 b	4.05 ± 0.17 b
Globulin (g/dl)	2.37 ± 0.18 a	0.87 ± 0.19 c	1.28 ± 0.12 b	1.32 ± 0.11 b	1.42 ± .15 b
A/G ratio	1.90 ± 0.2 c	4.05 ± 1.05 a	3.20 ± 0.2 ab	3.05 ± 0.25 b	2.85 ± 0.5 b

*AST* aspartate aminotransferase (u/l), *ALT* alanine aminotransferase (u/l), *ALP* alkaline phosphatise (u/l)

a, b, c means in the same row with different superscripts are significantly (*P* < 0.05) different

El-Afifi et al. [53] revealed that probiotics decreased the effect of AF on body weight gain and improved blood parameters, liver function, and renal function. Probiotic results support their findings. The blood’s hematology and biochemistry were enhanced (*P*<0.05) by all supplements (probiotic, ajowan, and probiotic + ajowan). Additionally, the liver and kidney functions, as well as serum biochemical markers and mouse weight gain, were all improved by *Saccharomyces cerevisiae* (SC) (P<0.05). However, it was discovered that SC was a secure and effective agent for reducing the toxicity of AFs and guarding against the toxicity that AFs caused [54]. According to Iram et al. [37], ajowan’s content may be the cause of its activation factor for AFs, which may explain why blood parameters improved [42, 55–67].

## Conclusions

The conclusion that can be derived from the findings mentioned above and discussion is that probiotic 3 (AVI-5-BAC) + ajowan can be added to rabbit diets to reduce and eliminate the toxicity of AFB1 and enhance growth performance criteria.

## Data Availability

Not applicable.

## Abbreviations

AFB_1_: Aflatoxins B_1._
AFM1: Aflatoxin M1
AFs: Aflatoxins
ALP: Alkaline phosphatase
ALT: Alanine aminotransferase
AST: Aspartate aminotransferase
CF: Crude fiber
DCP: Digestible crude protein
DM: Dry matter
DON: Deoxynivalenol
EE: Ether extract
EU: European Union
LAB: Lactic acid bacteria
NAGP: Non-antibiotic growth promoter
NFE: Nitrogen-free extract
NZW: New Zealand White
OM: Organic matter
OTA: Ochratoxin A
ROS: Reactive oxygen species
SC: *Saccharomyces cerevisiae*
TLC: Thin layer chromatography
ZEA: Zearalenone
